# Distinct Roles for Aryl Hydrocarbon Receptor Nuclear Translocator and Ah Receptor in Estrogen-Mediated Signaling in Human Cancer Cell Lines

**DOI:** 10.1371/journal.pone.0029545

**Published:** 2012-01-03

**Authors:** Mark P. Labrecque, Mandeep K. Takhar, Brett D. Hollingshead, Gratien G. Prefontaine, Gary H. Perdew, Timothy V. Beischlag

**Affiliations:** 1 Center for Molecular Toxicology and Carcinogenesis, Department of Veterinary and Biomedical Sciences, The Pennsylvania State University, Pennsylvania, United States of America; 2 The Faculty of Health Sciences, Simon Fraser University, Burnaby, Canada; French National Centre for Scientific Research, France

## Abstract

The activated AHR/ARNT complex (AHRC) regulates the expression of target genes upon exposure to environmental contaminants such as 2,3,7,8-tetrachlorodibenzo-*p*-dioxin (TCDD). Importantly, evidence has shown that TCDD represses estrogen receptor (ER) target gene activation through the AHRC. Our data indicates that AHR and ARNT act independently from each other at non-dioxin response element sites. Therefore, we sought to determine the specific functions of AHR and ARNT in estrogen-dependent signaling in human MCF7 breast cancer and human ECC-1 endometrial carcinoma cells. Knockdown of AHR with siRNA abrogates dioxin-inducible repression of estrogen-dependent gene transcription. Intriguingly, knockdown of ARNT does not effect TCDD-mediated repression of estrogen-regulated transcription, suggesting that AHR represses ER function independently of ARNT. This theory is supported by the ability of the selective AHR modulator 3′,4′-dimethoxy-α-naphthoflavone (DiMNF) to repress estrogen-inducible transcription. Furthermore, basal and estrogen-activated transcription of the genes encoding *cathepsin-D* and *pS2* are down-regulated in MCF7 cells but up-regulated in ECC-1 cells in response to loss of ARNT. These responses are mirrored at the protein level with cathepsin-D. Furthermore, knock-down of ARNT led to opposite but corresponding changes in estrogen-stimulated proliferation in both MCF7 and ECC-1 cells. We have obtained experimental evidence demonstrating a dioxin-dependent repressor function for AHR and a dioxin-independent co-activator/co-repressor function for ARNT in estrogen signalling. These results provide us with further insight into the mechanisms of transcription factor crosstalk and putative therapeutic targets in estrogen-positive cancers.

## Introduction

Elucidating the mechanisms underlying transcription is crucial to our understanding of how cells and organisms respond to physiological signals and environmental stimuli. The aryl hydrocarbon receptor (AHR) and the aryl hydrocarbon receptor nuclear translocator (ARNT) are members of the basic helix-loop-helix/PER-ARNT-SIM (bHLH-PAS) family of proteins and form a heterodimeric transcription factor upon binding a variety of environmental contaminants, including 2,3,7,8-tetrachlorodibenzo-*p*-dioxin (TCDD) [1]. The activated AHR/ARNT complex (AHRC) plays key roles in carcinogenesis and regulates the expression of *Cytochrome P4501A1* (*CYP1A1*) and other xenobiotic target genes to combat the effects of environmental contaminants [1]. In addition, AHR has been shown to be important for normal development and physiological homeostasis [2], [3], [4] and is essential for certain functions of the immune response, such as regulation of interleukin-17 producing T-helper cells [5] and induction of the cytokine interleukin-6 in MCF7 breast cancer cells [6].

Unliganded AHR exists in the cytoplasm as part of a multimeric complex containing two molecules of HSP90, the HSP90 co-chaperone p23, and hepatitis B virus X-associated protein 2 (XAP2) [7], [8], [9], [10]. Upon ligand binding, AHR translocates to the nucleus where it associates with ARNT to form a functional transcription factor complex, the AHRC. As an activated complex, the AHRC is capable of recruiting regulatory proteins, such as steroid receptor coactivator-1 (SRC-1), CREB binding protein (CBP/p300), NCoA2/GRIP1 [11], [12], receptor-interacting-protein 140 (RIP140) [13], CoCoA [14], GAC63 [15], NcoA4 [16] and TRIP230 [17], which play significant roles in determining the activity of TCDD-induced gene transcription. These co-activators and co-repressors incorporate themselves into multimeric complexes that modify chromatin structure, stabilize core transcriptional machinery, and mediate RNA chain elongation [18]. In addition to these classic transcriptional co-activators and co-repressors, AHR is recruited by other transcription factors during transcription, including estrogen receptor-α (ERα) [11], [19], [20] and NF-κB [21] to modify their intrinsic activities.

ERα/β are ligand activated transcription factors that belong to the superfamily of nuclear hormone receptors (NR) [22] and bind 17β-estradiol (E2) to regulate genes involved in reproduction and cellular growth and proliferation [23]. Upon ligand binding, ER forms a functional homodimer and binds its cognate response elements. Interestingly, there is a ligand-dependent reciprocal disruption between ER and AHR signaling. For instance, activated ERα inhibits AHRC activity at *CYP1A1* through direct protein-protein interactions, termed *transrepression* [11]. Conversely, TCDD's anti-estrogenic properties are well documented as it represses the E2-inducible genes *pS2* and *cathepsin-D (CAT-D)* [24], [25], [26]. However, the mechanisms of repression occurring at E2-responsive genes are unclear. Proposed theories for this repression include: (i) competition for a common pool of co-activators [27]; (ii) a direct down-regulation of *CAT-D* transcription through upstream inhibitory dioxin response elements [28]; (iii) activation of a TCDD-inducible inhibitory factor [28]; (iv) an AHR-dependent E3-ligase that degrades proteins crucial for ER-signalling [29], or; (v) a direct transrepression interaction between AHR and ER [11], [19].

In this study, we examined the role of AHR and ARNT on ERα-dependent target gene transcription in the human MCF7 breast cancer and the human ECC-1 endometrial-cervical cancer cell lines. Our data suggest that AHR and ARNT act independently from each other at off-target sites and we revealed that ARNT is not essential for TCDD-dependent repression of ER-signaling. In addition, we have demonstrated that ARNT acts as a cell specific coactivator in MCF7 cells, and as a corepressor in ECC-1 cells. Finally, we show that ARNT knockdown not only affects accumulation of mRNA and protein of ERα-target genes, but also has phenotypic consequences by influencing ERα-mediated cell proliferation.

## Results

### AHR-dependent repression of estrogen signaling in ECC-1 cells

**Figure 1:**
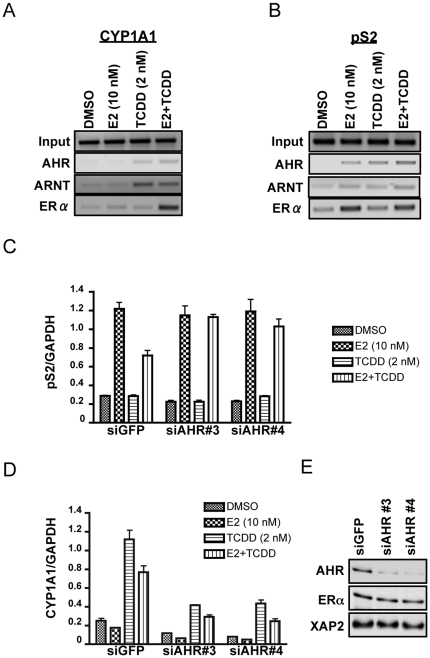
AHR is required for TCDD-mediated repression of E2-induced gene transcription. Chromatin immunoprecipitation assays of the *CYP1A1* enhancer (**A**) and *pS2* promoter (**B**) regions in ECC-1 cells using antibodies targeting AHR, ARNT or ERα. Cells were treated for 45 min with either DMSO, E2 (10 nM), TCDD (2 nM) or a combination of E2 and TCDD. (**C and D**) The functional role of AHR in TCDD-mediated transcription. ECC-1 cells were transfected with siRNAs to either GFP (siGFP) as a negative control or AHR (siAHR#3 or siAHR#4) 24 h prior to ligand treatment, then cells were treated with DMSO, E2 (10 nM), TCDD (2 nM) or a combination of E2 and TCDD. The mRNA levels for *pS2* (**C**) and *CYP1A1* (**D**) were determined through real-time RT-PCR and normalized to constitutively active GAPDH expression. (**E**) ECC-1 cells were transfected with siRNA's to AHR and harvested for whole cell lysates for Western Blot analysis of AHR, ERα and XAP2 protein levels.

The presence of AHR, ARNT and ERα at the dioxin-inducible *CYP1A1* enhancer and the E2-inducible *pS2* promoter has already been documented in MCF7 cells and other breast cancer cell lines [11], [19], [20], [30] . Although preliminary studies have identified ECC-1 human endometrial cells as an ideal system to study dioxin disruption of estrogen signaling [31], [32] very little is known concerning the roles of AHR, ARNT and ER and their respective interactions in this cell line. We employed the ChIP assay to ascertain the status of these proteins at the *pS2* promoter and *CYP1A1* enhancer in both the presence and absence of E2 and TCDD. ECC-1 cells were treated with DMSO, 10 nM E2, 2 nM TCDD or a combination of E2 and TCDD for 45 min. After chemically cross-linking protein to DNA with formaldehyde, cells were harvested and sonicated. Sheared DNA-protein complexes were precipitated with antibodies specific to AHR, ARNT or ERα and then isolated complexes were reverse-crosslinked and DNA was subjected to PCR amplification. Consistent with other investigators' observations in breast cancer cells, we observed the recruitment of AHR and ARNT on the human *CYP1A1* enhancer in a TCDD-dependent fashion and ERα was greatly enriched only after treatment with a combination of 2 nM TCDD and 10 nM E2 (Figure 1A). Furthermore, the recruitment of ERα to the *pS2* promoter occurs in an E2-dependent fashion while AHR and ARNT are present after treatment with either ligand but are enriched during co-treatment (Figure 1B).

We have previously shown that ERα associates with the AHRC to mediate estradiol-dependent transrepression of dioxin-inducible gene transcription [11]. Therefore, we set out to determine the functional significance of AHR in estrogen-inducible gene transcription. We transfected ECC-1 cells with small inhibitory RNA directed towards either AHR (siAHR) or a GFP negative control (siGFP). After transfection, cells were serum starved for 24 h, followed by 24 h ligand treatment and then harvested for total mRNA that was quantified through quantitative PCR. As expected, ablation of AHR and co-treatment with 2 nM TCDD and 10 nM E2 results in the loss of TCDD-induced repression of *pS2* transcription (Figure 1C). However, loss of AHR had no measurable effect on basal, or estrogen activated *pS2* mRNA accumulation. As a control for siRNA specificity, Western blots of AHR protein show a greatly reduced expression of AHR protein after transfection and unchanged protein levels of ERα and XAP2 which served as a loading control (Figure 1E). These demonstrate the specificity of the siRNA's to AHR, and that loss of AHR does not affect accumulation or turnover of basal ERα protein levels. Furthermore, the induction of *CYP1A1* transcription with TCDD after AHR knockdown is attenuated when compared to the siGFP negative control (Figure 1D). Thus, AHR is a requirement for TCDD-induced repression of E2-responsive gene transcription and the increased transcriptional response with combinatorial treatment is due solely to the loss of AHR and other putative transcriptional modifiers associated with its presence. Finally, we obtained essentially identical results in both MCF7 and ECC-1 cell lines under serum-starved or charcoal-stripped serum conditions suggesting that differences in the cell lines' ability to go through cell cycle does not impact this phenomenon. Together, these results support the concept that the ECC-1 cell line is an excellent alternative cell model to study dioxin-induced disruption of estrogen receptor signaling.

### ARNT has cell specific co-activator/co-repressor functions

**Figure 2:**
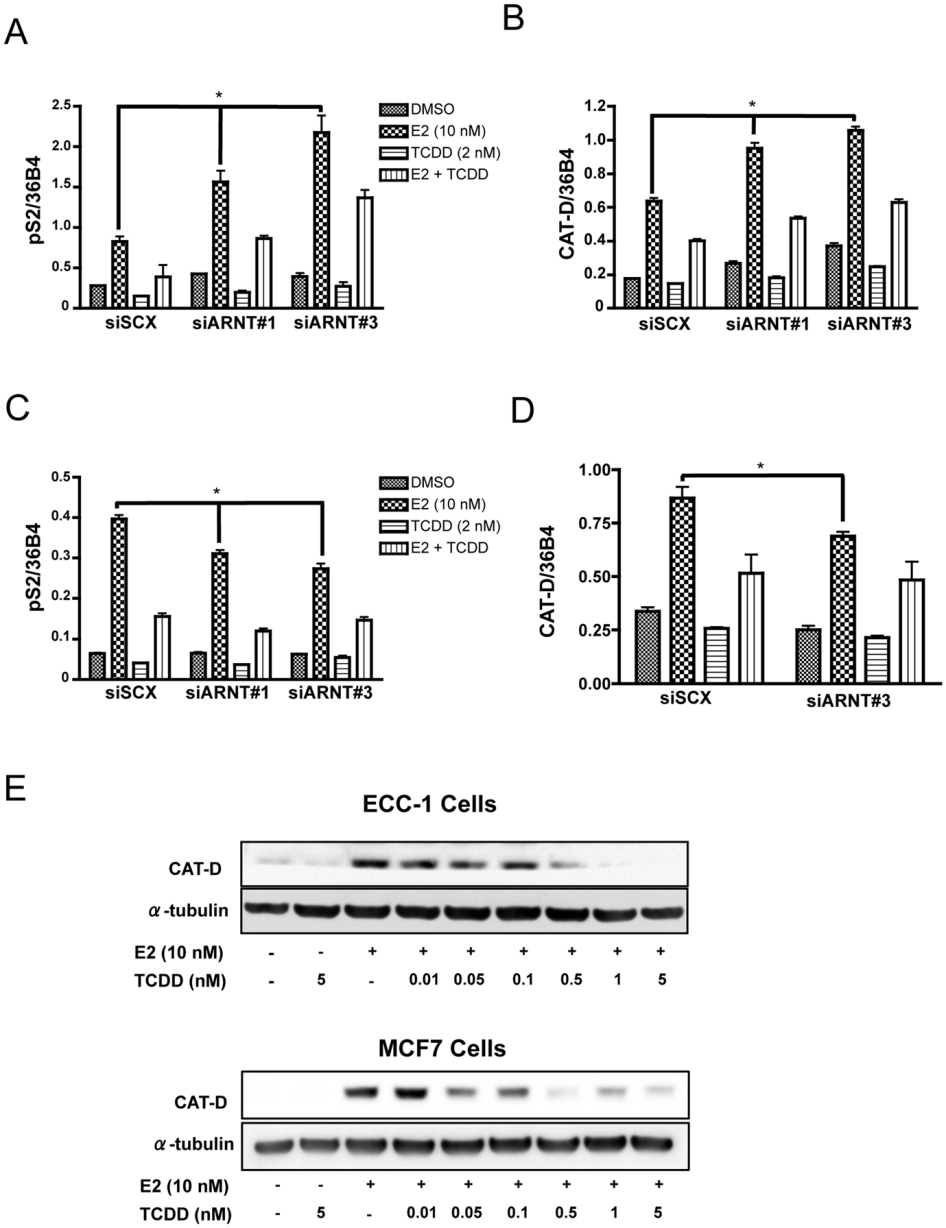
Loss of ARNT shows a cell specific co-activator or co-repressor transcriptional function. ECC-1 cells (**A and B**) and MCF7 cells (**C and D**) were transfected with either scrambled siRNA (siSCX) or siRNA directed to ARNT (siARNT#1 or siARNT#3). Twenty-four hours after transfection, cells were treated with vehicle (DMSO), E2 (10 nM), TCDD (2 nM) or a combination of E2 and TCDD. Gene expression was determined by real-time RT-PCR after isolation and reverse transcription of total RNA. *CAT-D* and *pS2* expression were normalized to constitutively active 36B4 gene expression. (**E**) Western blot analysis of CAT-D protein levels in MCF7 and ECC-1 cells. Cells were treated with DMSO or E2 (10 nM) and varying concentrations of TCDD (10 pM, 50 pM, 100 pM, 500 pM, 1 nM or 5 nM). After 24 hours of treatment, whole cell lysates were harvested, and Western Blot assays were performed using antibodies directed against CAT-D and α-tubulin. Error bars represent ± S.D. * p<0.05.

The identification of ARNT as a co-activator in estrogen signalling [27], [30], juxtaposed with the known transrepressor effects of TCDD, led us to investigate the role played by ARNT in TCDD-mediated transrepression of ER function in various human cancer cell lines, namely MCF7 and ECC-1 cells. Through siRNA directed towards ARNT and a scrambled negative control (siSCX), and subsequent qPCR analysis of endogenous *pS2* and *CAT-D* gene transcription, we made several interesting observations. First, ARNT displays co-repressor properties in ECC-1 cells. Accumulation of *pS2* (Figure 2A) and *CAT-D* (Figure 2B) mRNA levels are exacerbated during E2 treatments after loss of ARNT suggesting a cell specific function for ARNT independent of AHR. Furthermore, we observed this phenomena with three separate siRNAs directed towards ARNT (siRNA's 1 and 3 are shown in Figure 2A, B and C). We used at least two siRNA's for all other experimental parameters with essentially identical results, but for brevity, hereafter we only present data depicting the use of one siRNA. Secondly, consistent with the findings of several other investigators, ARNT displayed co-activator properties in MCF7 cells [27], [30]. Indeed, knockdown of ARNT protein dampens E2-induced transcription of *pS2* (Figure 2C) and *CAT-D* (Figure 2D). Finally, in a dose response experiment, we observed a concomitant decrease of CAT-D protein levels with increasing concentrations of TCDD in both ECC-1 and MCF7 cells (Figure 2E). These data indicate that ARNT function as it relates to ER signaling is likely dictated by other factors specific to the cellular environment.

**Figure 3:**
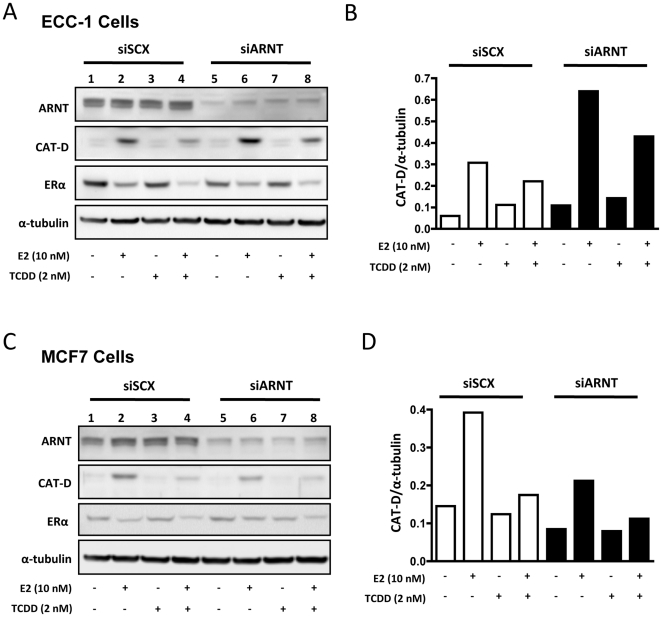
Effect of ARNT knockdown on ARNT, CAT-D and ERα protein levels. ECC-1 (**A and B**) and MCF7 (**C and D**) cells were transfected with either siSCX or siARNT and ligand treated as described in Figure 2. (**A and C**) Representative Western blots of ARNT, CAT-D, ERα and α-tubulin protein levels. Bar graphs of CAT-D protein levels in ECC-1 (**B**) and MCF7 (**D**) cells after normalizing luminescence values to α-tubulin. Open bars represent ligand treatments after siRNA to scrambled negative control (siSCX) and closed (black) bars represent ligand treatments after siRNA to ARNT (siARNT). Experiments were performed three times with essentially identical outcomes.

To determine if the modulation of transcriptional activity translated into similar protein expression profiles, we used Western blot analysis to visualize the level of CAT-D protein after ARNT knockdown. Cells were transfected and ligand treated under identical conditions as the qPCR parameters. When compared to the scrambled negative control, CAT-D protein expression after E2 treatment is exacerbated in ECC-1 cells with ARNT knockdown (Figure 3A). Conversely, MCF7 cells transfected with ARNT siRNA resulted in a blunted expression of CAT-D protein during E2 treatment (Figure 3C). Furthermore, ERα levels remained consistent, regardless of ARNT status (Figure 3A and C). Most importantly, dioxin-induced repression of ER signaling was maintained at the protein level after ARNT knockdown (Figure 3A and C). The representative blots were normalized to α-tubulin and luminescence was measured using GeneTools 4.01.2 software (Syngene). These normalized values showed a two-fold induction of CAT-D in ECC-1 cells with ARNT knockdown after E2 treatment (Figure 3B, columns 2 and 6), whereas in MCF7 cells, knock-down of ARNT resulted in a 40% decrease in E2-dependent CAT-D expression (Figure 3D, colunms 2 and 6). These results provide concrete evidence that the level of protein expression mirrors the transcriptional response in both cell lines and that physiological consequences must ensue with loss of ARNT.

**Figure 4:**
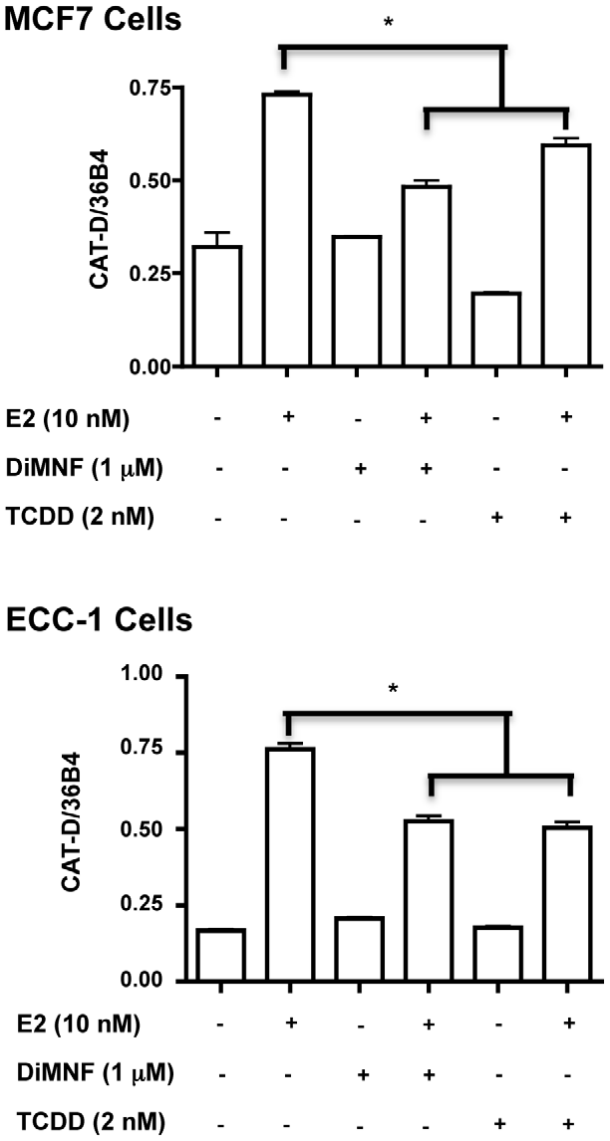
Effect of selective aryl hydrocarbon receptor modulators on estrogen-inducible transcription. The effect of 1 µM DiMNF on E2-inducible CAT-D expression in MCF7 and ECC-1 cells. Cells were treated with the indicated ligands for 24 h prior to RNA isolation. Gene expression was determined as described above. Error bars represent ± S.D. * p<0.05.

These surprising results were coupled with the observation that TCDD-induced repression of ER-signaling is maintained in both cell lines after ARNT knockdown (Figure 2 and 3) thus providing evidence that ARNT is not required for TCDD/AHR-dependent repression of ERα signaling. This hypothesis is supported by the ability of a selective aryl hydrocarbon receptor modulator (SAHRM) to repress estrogen signaling. We examined the ability of the known SAHRM, DiMNF [33], [34] to repress E2-mediated transcription by RT-PCR in MCF7 and ECC-1 cells (Figure 4). DiMNF was as effective at repressing CAT-D mRNA accumulation as TCDD. DiMNF is a potent antagonist of AHR and effectively displaces TCDD from the receptor at 1 µM [34]. In addition, DiMNF fails to induce AHR-ARNT-dioxin response element formation in an electrophoretic mobility shift assay, suggesting that AHR-ARNT dimerization may not occur [34], thus, it seems likely that AHR's transrepression function is entirely ARNT-independent.

### ARNT knockdown causes increased proliferation in ECC-1 cells and decreased proliferation in MCF7 cells

**Figure 5:**
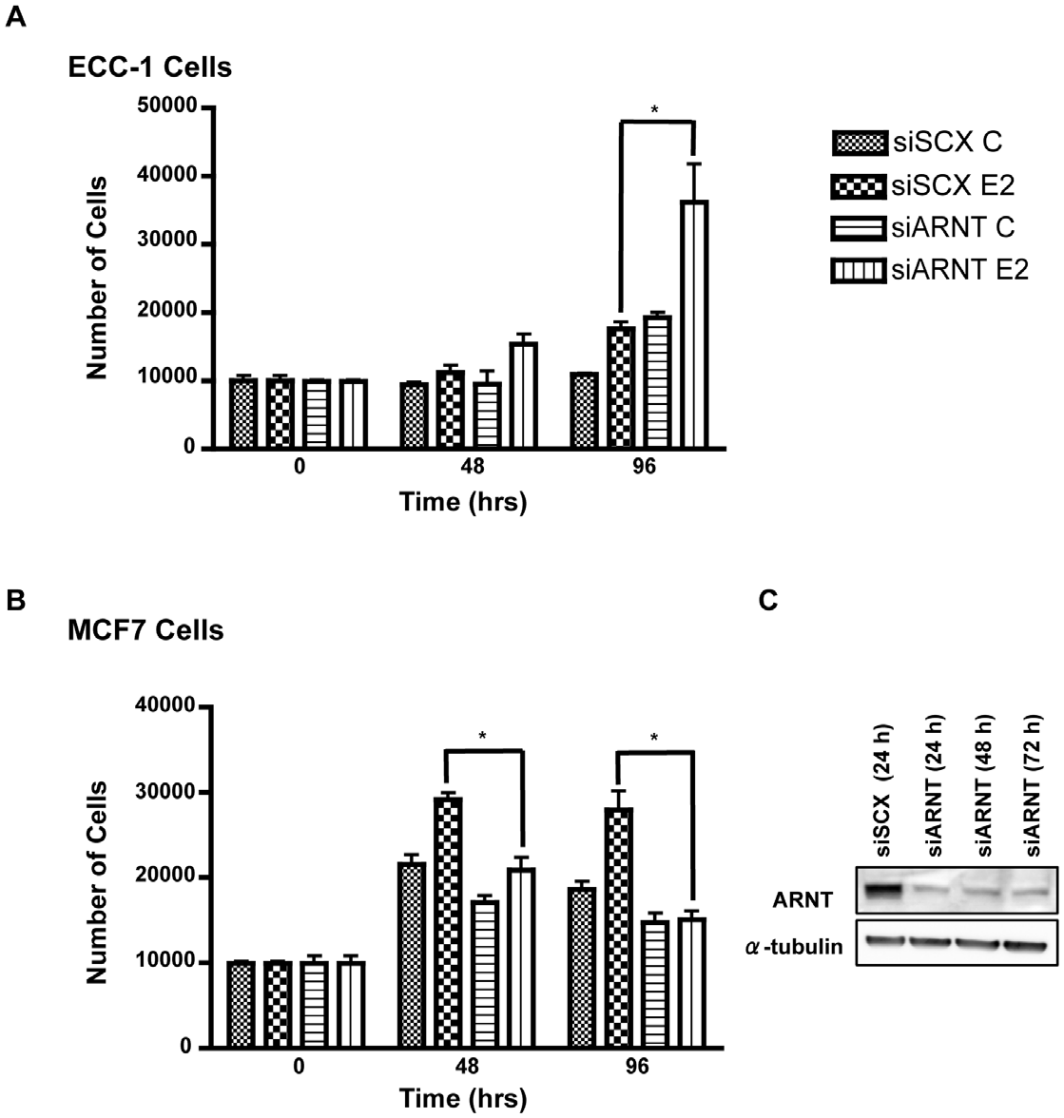
Loss of ARNT promotes cell proliferation in ECC-1 cells and attenuates growth in MCF7 cells. ECC-1 (**A**) and MCF7 (**B**) cells were transfected with either siSCX or siARNT for 6 hours, then trypsinized and reseeded at 10,000 cells/well in 12-well dishes. Twenty-four hours after seeding, cells were treated with either DMSO or E2 (10 nM) and cell counts were conducted at 0, 48 and 96 hours after treatments with a Fuchs-Rosenthal Counting Chamber. At the 48 h time point, cells were treated a second time with 10 nM E2. Experiments were done in triplicate and each trial was counted three times. (**C**) Western blot analysis of ARNT after siRNA knock-down reveals that significant knock-down was achieved and persists over a 72 h period. Error bars represent ± S.D. * p<0.05.

Sensitivity to estrogen has been linked to proliferation and cell transformation in ER-positive carcinoma cells [35]. To establish if ARNT can influence E2-dependent cellular proliferation, we performed proliferation assays on ECC-1 and MCF7 cells after ARNT siRNA treatment. Consistent with our quantitative PCR and Western blot data, ECC1 and MCF7 cells displayed altered rates of proliferation after ARNT knockdown compared to cells transfected with the scrambled negative control siRNA (Figure 5A and B). Knockdown of ARNT was monitored for 72 h and the significant knock-down observed was unchanged essentially at each time point (Figure 5C). Neither cell line displayed altered growth patterns until the 48 hour time point. At 48 hours, ECC-1 cells in both the siSCX and siARNT conditions showed modest proliferation in response to E2 treatments. At the 96 hour time point, there was a highly significant increase in E2-inducible proliferation of ECC-1 cells treated with siARNT, compared to scrambled control treated cells. Intriguingly, ARNT knockdown increased basal growth rates and caused an exacerbated response to E2 with the cell number nearly doubling in the siARNT E2 treatments compared to the siSCX E2 treatments. Conversely, MCF7 cells showed a decreased proliferation rate after ARNT knockdown (Figure 5B). The growth rates were not significantly different until the 48 h time point, when the E2-inducible proliferative response in the scrambled negative control was apparent. The siARNT transfected cells had a blunted growth response during both control and E2 conditions. At 96 hours, the cells began losing sensitivity to E2 and entered into a senescent state. Whether or not this was due to the growth conditions is unclear. However, these data further support the hypothesis that ARNT has co-repressor properties in ECC-1 cells and co-activator properties in MCF7 cells. Moreover, the sensitivity to E2 and the reciprocal growth responses exhibited by the two cell lines are bona fide phenotypic consequences of ARNT ablation.

## Discussion

Many environmental contaminants that serve as activators of the aryl hydrocarbon receptor are known as putative endocrine disrupting compounds. Exposure to many of these compounds occur on a daily basis and represents significant risks to human health. In particular, the repressive effects elicited by ligands of the AHR on ER signaling have been well documented [11], [19], [36], [37], [38]. Other reports have described the co-activator potential of ARNT for ER-mediated transcription [27]. Despite the growing body of evidence to support roles for AHR and ARNT in ER function, little is known about the normal physiological role of these proteins as they relate to estrogen signaling or the molecular determinants of toxicant-induced transrepression of ER function by AHR. Finally, this investigation revealed that ARNT is not essential for AHR-mediated off-target transrepression. In order to delineate the molecular mechanisms underlying AHR-mediated transrepression we sought to uncouple AHR and ARNT function in ER-positive human cancer cell lines. In doing so, we discovered that ARNT attenuates activated ER-target gene transcription in ECC-1 cells, in direct contrast to its co-activator function described in MCF7 cells [27].

**Figure 6:**
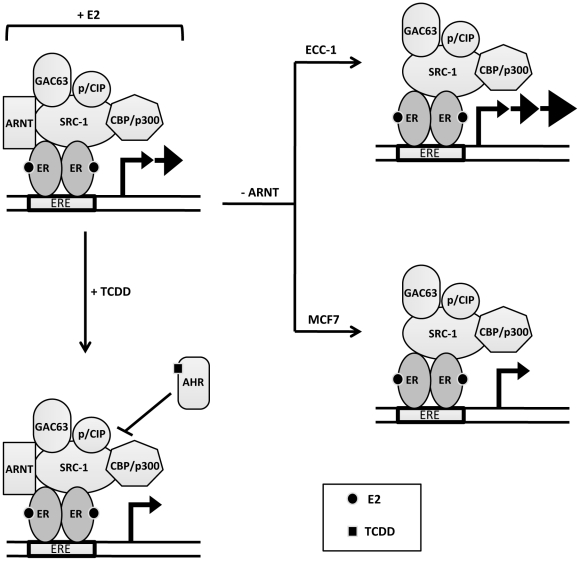
A schematic representation of estrogen signaling, describing the transcriptional functions of ER, AHR and ARNT in ECC-1 and MCF7 cells. The presence of E2 facilitates the assembly of transcriptional modifiers that induce transcription of E2-responsive genes. Ligand activated AHR represses ER-signaling independent of ARNT. The loss of ARNT in ECC-1 cells, where it acts as a co-repressor, leads to increased sensitivity to E2 and increased transcriptional activity at ER regulated genes. The loss of ARNT in MCF7 cells, where it acts as a co-activator, leads to decreased sensitivity to E2 and decreases transcriptional activity at ER regulated genes.

ARNT was originally identified as a bona fide transcription factor [39] and dimerization partner of AHR [40]. Beyond its canonical transcription factor function, ARNT can interact with several other transcription factors, including ERα [11] and its co-activator function for ER signaling has been well described [27], [30]. Therefore, we expected that knockdown of ARNT in ECC-1 endometrial-cervical cancer cells would result in a diminution of ER target gene expression. The resulting increase in mRNA accumulation, protein expression and E2-inducible proliferation strongly suggests that ARNT acts as a transcriptional co-repressor in this cell line. The molecular mechanism(s) underlying the differences observed in MCF7 and ECC-1 cells likely is related to differences in the nature and composition of the ancillary transcriptional machinery recruited by ARNT in each cell line. ARNT presents several different protein-protein interaction domains for the recruitment of co-activator proteins, including a carboxy-terminal transactivation domain [18], its PAS-B region [41] and its basic-helix-loop-helix domain [12]. In addition, an association between ARNT and the transcriptional co-repressor protein, SMRT has been demonstrated [42]. However, we believe that this is the first demonstration that ARNT has dual co-activator/co-repressor functions in a cell-specific fashion (Figure 6). In addition, the repercussions of these transcriptional effects can be observed at the translational and phenotypic levels.

Several models have been hypothesized to explain the molecular mechanisms underlying transcription factor mediated cross-talk, particularly the ability of one transcription factor to repress another's function [18]. Reugg and colleagues, among others, have suggested that transcription factor-mediated transrepression might be the result of competition for a limited pool of co-activator proteins [27]. However, the identification of ARNT as a co-repressor in ECC-1 cells unmasks a more complex mechanism of transrepression at E2-inducible genes then the co-activator competition model suggests. While these data do not definitively disprove this model, we believe that our data suggest that it is unlikely because co-activator competition cannot account for TCDD-inducible repression in ECC-1 cells as activated AHR should squelch ARNT's co-repressor function. In addition, ARNT, a protein that has demonstrated the ability to recruit numerous co-activators [12], [15], [17], [41], [43], [44] should illicit an effect similar to ligand-activated AHR if co-activator pools were in such limited supply that competition would hinder individual transcription factor function. This clearly is not the case in the ECC-1 cell line.

The experimental evidence presented above indicates that AHR does not require ARNT to mediate its off-target transrepressor effects. Loss of ARNT in both MCF7 and ECC-1 cells failed to abrogate the repressive effects of TCDD on E2-inducible transcription and protein expression (Figures 2 and 3). The effects DiMNF-bound AHR are independent of dioxin response element binding as nuclear extract from cells treated with DiMNF does not retard the movement of a labeled dioxin response element probe in gel shift assays [34]. Furthermore, AHR-ARNT dimerization is a requirement for DNA binding, suggesting that this may not occur in the presence of DiMNF. This represents a paradigm shift in our understanding of AHR function and has implications for the use and effectiveness of selective AHR modulators. Indeed, a novel AHR antagonist has proven to be a potent stimulator of AHR-dependent stem cell expansion [45]. Thus, antagonists that do not elicit AHR-ARNT dimerization might be more effective repressors of ER function than pure agonists as AHR would not be squelched by ARNT binding. The SARHM DiMNF is a potent AHR-dependent repressor of cytokine signaling [33], [34]. Furthermore, DiMNF was effective in repressing ER-regulated target gene expression (Figure 4). Molecular modelling studies demonstrate that DiMNF forms an extra hydrogen bond with AHR at Thr289 [34], a characteristic not shared with the partial agonist α-naphthoflavone suggesting that the DiMNF-AHR adopts a unique confirmation. Thus, the flavonoids represent a class of compounds that may be attractive targets for further testing and development to determine their effects on ER target gene expression.

Our experimental evidence demonstrates a TCDD-dependent repressor function for AHR and a TCDD/AHR-independent co-activator/co-repressor function for ARNT in estrogen signaling. These results provide us with further insight into the mechanisms of transcription factor crosstalk and putative therapeutic targets in estrogen-positive cancers. Taken together, our data suggest a more complex mechanism of ARNT function and AHR-mediated transrepression of ER-signaling than previously suggested. The clinical utility of these findings remains to be tested and will be the subject of future investigations.

## Materials and Methods

### Materials and Cell culture

3′,4′-dimethoxy-α-naphthoflavone (DiMNF) was obtained commercially (Indofine Chemical Co., Hillsborough, NJ). ECC-1 and MCF7 cells (ATCC) were maintained in Dulbecco's Modified Eagle's Medium (DMEM; BioWhittaker, Lonza,) with 10% fetal bovine serum (FBS; HyClone, PerBio, Thermo Fisher Scientific Inc.) and supplemented with 100 units/ml potassium penicillin-100 µg/ml streptomycin sulphate (BioWhittaker, Lonza) at 37°C, 20% O_2_, and 5% CO_2_. Twenty-four h before any experimental perturbation, cells were washed 2× with PBS, and maintained in Phenol-Red-free media without FBS [46].

#### Chromatin immunoprecipitation assays

Chromatin immunoprecipitation (ChIP) assays were performed as described by previously [17]. Briefly, cells were plated into 150 cm^2^ dishes and serum-starved 24 h before treatment. Treatment of cells was done in serum-free media supplemented with 3 mg/mL bovine serum albumin for 45 min. Chromatin complexes were chemically cross-linked using a 1% formaldehyde/0.7 mol/L HEPES solution (final concentration), pH 7.8, and complexes were sonicated to yield DNA fragments of 200 to 900 bp size. Complexes were precleared with protein A agarose resin (CalBiochem) and incubated overnight with specific antibodies [ERα rabbit polyclonal or ARNT goat polyclonal (Santa Cruz) or AHR rabbit polyclonal described previously [47]. Immunoadsorbed complexes were captured on protein A agarose resin and washed twice with 0.5× RIPA, followed by three washes with 10 mmol/L Tris-HCl (pH 8.0) and 1 mmol/L EDTA. Samples were eluted off of the resin using 100 mmol/L NaHCO3 and 1% SDS, and cross-links were reversed at 65°C overnight. Samples were phenol-chloroform extracted and precipitated with 70% EtOH and Pellet Paint (Novagen). Immuno-adsorbed DNA was analyzed by PCR. Primers for the *CYP1A1* enhancer and pS2 promoter have been described previously [20], [48].

### Transient transfections

MCF7 and ECC-1 cells were cultured in the conditions described above until approximately 70% confluent before siRNA transfection. Cells were transfected with either Green Fluorescence Protein (GFP) siRNA (Dharmacon), scrambled (SCX) siRNA (DS Scrambled negative control siRNA, Integrated DNA Technologies Inc.), AHR siRNAs (Dharmacon) or ARNT siRNAs (Integrated DNA Technologies Inc., Cat. No. HSC.RNAI.N187426.11.1, HSC.RNAI.N178426.11.2, HSC.RNAI.N178426.11.3; siARNT 1, siARNT 2, and siARNT 3 respectively). Cells were transfected with 10–15 nM siRNA using 0.3% (v/v) Trifectin (Integrated DNA Technologies) according to manufacturer's protocol. The cells were allowed to incubate in transfection mix for 6 h at 37°C, and 5% CO_2_ after which the transfection mix was removed and replaced with serum free medium.

### Reverse transcription and Real-Time PCR

Reverse transcription and real-time PCR were performed as described previously [11]. In brief, cells were treated either with DMSO (Me_2_SO), TCDD (2 nM), E2 (10 nM), or a combination of TCDD and E2, for 24 h. For studies involving the selective AHR modulator 1 µM 3′,4′-dimethoxy-α-naphthoflavone (DiMNF) with either DMSO, E2 (10 nM), diMNF (1 µM) or E2 and diMNF in combination. Cells were harvested in TRI Reagent (Sigma) and total RNA was isolated and subjected to reverse transcription using a High Capacity cDNA Archive kit (Applied Biosystems). Complimentary DNA was amplified by real-time PCR using a Power SYBR Green PCR kit (Applied Biosystems) according to manufacturer's protocols. Oligonucleotide pairs used to amplify human cDNA sequences were described previously [11]. DNA was amplified for 45 cycles in a StepOne Plus Real-Time PCR System (Applied Biosystems).

### Western blot analysis

In order to determine the effects of TCDD on E2-inducible protein levels, MCF7 and ECC-1 cells were treated either with vehicle (Me_2_SO), TCDD (2 nM), E2 (10 nM), or a combination of TCDD and E2 for 24 h. For protein analysis that included siRNA treatment, cells were transfected for 6 h and then starved in serum free media for 24 h prior to ligand treatments. Cells were harvested and the protein concentration determined by the RC DC protein assay (Bio-Rad). Equal amounts of proteins from the samples were resolved on a SDS-acrylamide gel then transferred to polyvinylidene fluoride (PVDF) membrane. Upon completion of transfer, the membrane was wetted with 100% methanol then probed with anti-ARNT (goat polyclonal IgG; Santa Cruz Biotechnology, Inc.), anti-AHR (rabbit polyclonal, Biomol, GmbH), anti-CAT-D (rabbit polyclonal IgG; Santa Cruz Biotechnology, Inc.), anti-ERα (rabbit polyclonal IgG; Santa Cruz Biotechnology, Inc.), anti-α-tubulin (mouse monoclonal IgG; Santa Cruz Biotechnology, Inc.) or anti-XAP2 mouse monoclonal antibody. The detection was performed using horseradish peroxidase conjugated anti-mouse or anti-rabbit or anti-goat IgG and ECL detection kit (GE Healthcare).

### Proliferation Assay

MCF7 cells and ECC-1 cells at 75–80% confluency were transfected with either ARNT or scrambled negative control siRNA. After 6 h, cells were washed 2 times with PBS, trypsinized and seeded into 12-well plates at 10,000 cells/well in DMEM with 2.5% charcoal-stripped FBS. Twenty-four hours after plating, E2 was added directly to half the wells to a final concentration of 10 nM. Cells were counted at; 0 (control), 12, 24, 48 and 96 h following E2 administration. A second treatment of E2 (10 nM) was added to the cells at 48 hrs. Determinations were performed in triplicate and each sample was counted three times.

### Statistical analysis

Statistical analyses were performed using GraphPad Prism 4.0. For multiple comparisons (i.e. siRNA experiments) statistical significance was determined using a 2-way ANOVA with Tukey's Multiple Comparison test. Values are presented as means ± standard error of the mean (SEM). A P value<0.05 was considered to be significant.
